# Modeling the Optimal Transportation for Acute Stroke Treatment: Impact of Diurnal Variations in Traffic Rate

**DOI:** 10.1007/s00062-020-00933-y

**Published:** 2020-07-16

**Authors:** Marielle Ernst, Marios-Nikos Psychogios, Eckhard Schlemm, Jessalyn K. Holodinsky, Noreen Kamal, Thomas Rodt, Henning Henningsen, Christoffer Kraemer, Götz Thomalla, Jens Fiehler, Caspar Brekenfeld

**Affiliations:** 1 Department of Diagnostic and Interventional Neuroradiology, University Medical Center Hamburg-Eppendorf, Haus Ost 22 (O 22), Martinistr. 52, 20246 Hamburg, Germany; 2 Department of Neuroradiology, Clinic for Radiology & Nuclear Medicine, University Hospital Basel, Basel, Switzerland; 3 Department of Neurology, University Medical Center Hamburg-Eppendorf, Hamburg, Germany; 4 Sunnybrook Research Institute, Sunnybrook Health Sciences Centre, Toronto, Ontario Canada; 5 Department of Industrial Engineering, Dalhousie University, Halifax, Canada; 6 Department of Diagnostic and Interventional Radiology, Klinikum Lüneburg, Lüneburg, Germany; 7 Department of Neurology, Klinikum Lüneburg, Lüneburg, Germany

**Keywords:** Stroke management, Endovascular therapy, Treatment effectiveness, Transportation time, Catchment area

## Abstract

**Purpose:**

Prolonged transfer times between the primary stroke center (PSC) and the comprehensive stroke center (CSC) are one of the major causes of treatment delay for endovascular stroke treatment. We aimed to analyze the effect of the diurnal variations in traffic rates at weekdays and weekends on the catchment area size of three transportation paradigms, i.e. mothership, drip-and-ship (DS) and drip-and-drive (DD).

**Methods:**

A conditional probability model that predicts the probability of good outcome for patients with suspected large vessel occlusion was used to analyze the prehospital stroke triage in northwest Germany and produce catchment area maps. Transportation times were calculated during each hour of a weekday and a Sunday using Google Maps. For comparison, real DD transportation times from our CSC in Hamburg-Eppendorf (blinded for review) to a PSC in Lüneburg were prospectively recorded.

**Result:**

On weekdays, the mothership catchment area was the largest (≥40,000 km^2^, 63%) except for a decrease during morning rush hours, when the DD catchment area was highest (30,879 km^2^, 48%). The DS catchment area was higher than the DD catchment area during the afternoon rush hours both during the week as well as on Sundays.

**Conclusion:**

Our study showed a considerable impact of the diurnal variations in traffic rate and direction of travel on optimal stroke transportation. Stroke systems of care should take real time traffic information into account.

**Electronic supplementary material:**

The online version of this article (10.1007/s00062-020-00933-y) contains supplementary material, which is available to authorized users.

## Introduction

Treatment options for patients with acute ischemic stroke include intravenous thrombolysis (IVT) and endovascular therapy (EVT). As the treatment effects of both IVT and EVT diminish over time[1, 2], stroke systems of care across the globe are currently faced with the challenge of determining the optimal transport destination for patients with suspected stroke.

Three triage strategies for patients with suspected large vessel occlusion (LVO) have been proposed: (1) the drip-and-ship (DS) paradigm, where patients are first transported to the nearest primary stroke center (PSC) to minimize time to IVT and then transferred to the nearest comprehensive stroke center (CSC) if they are a candidate for EVT, (2) the mothership paradigm, where patients are transported directly to a CSC, potentially bypassing a closer PSC and (3) the drip-and-drive (DD) paradigm, where the neurointerventionalist capable of performing EVT is transported to the stroke patient with proven LVO in the PSC. While there has been much prior discussion surrounding optimization of within hospital work flow,[3–6] one of the major causes of treatment delay is the prolonged transport times of stroke patients to and between hospitals [7–11].

Previously, conditional probability modeling was used to determine under what conditions the DD paradigm would predict the greatest probability of good outcomes for patients with suspected ischemic stroke due to LVO [12]. This model took into consideration the probability of an underlying LVO, the distance to the nearest PSC or CSC and the transfer time; however, the effect of changing traffic activity over the course of the day on the best transport option has yet to be modeled.

We aimed to model the effect of the diurnal variations in traffic patterns on weekdays and weekends on the catchment area size of the three transportation paradigms. Moreover, we present the number of emergency operations as well as transportation times across the day on weekdays and weekends for a DD cooperation between our CSC and a PSC with a transfer distance of 68 km.

## Material and Methods

The data that support the findings of this study are available from the corresponding author on reasonable request.

### Terminology

For the purposes of this study, a PSC is a hospital that provides IVT 24 h a day, 7 days per week and has in-house angiosuite staff and the necessary equipment to provide EVT but does not have a neurointerventionalist capable of EVT locally available. Radiologists and technicians at the PSC are trained in advanced procedures and materials used during emergency operations.

A CSC is a hospital that provides both IVT and EVT 24 h a day, 7 days per week. A CSC+ is a university hospital where >200 EVTs and >400 other neurointerventions are performed annually. It is adequately staffed to allow one of the neurointerventionalists capable of EVT to drive to the PSC to perform emergency operations in the DD paradigm. The car of the neurointerventionalist is equipped with flashing warning lights and sirens and can break traffic laws (such as going through red lights) as if it were an ambulance. The neurointerventionalists covering the on-call service for emergency operations performed at the PSC are familiar with the angiography suites of the PSC, as well as with the teams of the radiology, anesthesiology, and neurology departments.

### Real Times

A formal collaboration with our university hospital CSC+ in Hamburg-Eppendorf (blinded for review) was established to provide emergency operations at a PSC in Lüneburg which is 68 km away. All consecutive patients admitted between April 2016 and January 2020 to the PSC in Lüneburg with acute ischemic stroke due to LVO who were treated with EVT were included in this analysis. The time from the initial alert of the CSC+ about the patient to the arrival of the neurointerventionalist at the angiosuite of the PSC in Lüneburg (transfer time) and the date were prospectively recorded.

### Models

A previously published conditional probability model which predicts the probability of good outcome (modified Rankin scale score of 0–1 at 90 days post-stroke) for patients with suspected LVO was used [12, 13]. The model is based on the decrease in good outcome rates in EVT and IVT over time and also incorporates the geography, the probability of LVO, hospital efficiency and the time from onset to treatment to predict the probability of good outcome in the mothership, DS and DD paradigms. Details on model components have been previously published [12].

### Territory

Prehospital stroke triage was analyzed in the federal states of Hamburg, Schleswig-Holstein, and Lower Saxony in northwest Germany. The area covers 64,065 km^2^ with 21 PSCs, 23 CSCs, and 5 CSC+. This area has a population of 12,703,561 people (2017), 21.5% of whom are older than 65 years of age. In this region, the average distance between PSC and CSC/CSC+ (for DS transport) is 44 km and the average distance between CSC+ and PSC (for DD transport) is 67 km.

### Modeling Scenarios

The assumed baseline patient population had a score of ≥5 on the Rapid Arterial oCclusion Evaluation (RACE) scale and would thus screen positive for a probable LVO [14].

We assumed that 90% of LVO patients would be treated with EVT and that 80% of patients with LVO or non-LVO occlusions with onset-to-treatment times <4.5 h would receive IVT.

Based on proposed quality measures [3, 4, 15] and on our own experience the following time parameters were used: onset to first medical response time: 30 min, ambulance on scene time: 30 min, door to needle time at PSC: 30 min, needle to door time at PSC: 20 min and door to needle time at CSC and CSC+: 30 min. Door to groin puncture time at CSC/CSC+ was 60 min; however, if a transferred (drip and ship) patient arrived at the CSC/CSC+ within 90 min of first imaging at the PSC, it was assumed that the patient did not require reimaging and was transferred directly to the angiosuite, thus door to groin puncture time was only 30 min. Based on our own experience in the DD paradigm the time from IVT start (at the PSC) to the interventionalist leaving the CSC+ (needle to neurointerventionalist leave time) was 10 min and the time from neurointerventionalist arrival at the PSC to groin puncture was 20 min.

In previously published modelling work from this region [12] all modelling scenarios were based on the traffic conditions at 3 o’clock in the morning in order to simulate low traffic scenarios and ambulance right of way.

In this study, to simulate the traffic patterns across the day from the urban to the suburban area, we calculated the mean transportation time from our CSC+ in Hamburg-Eppendorf (blinded for review) to the PSC in Lüneburg (68 km distance) hourly over a 24 h period on a Tuesday and a Sunday to simulate traffic patterns on weekdays and weekends, respectively, using Google Maps (Google, Mountain View, CA, USA). The differences between these hourly drive times and those at 3 o’clock in the morning were calculated to determine the magnitude of the delay or acceleration in transport time across the day (Figure I in the online only data supplement). The differences found for this 68 km transport were generalized to all suburban to urban area transports.

For DD transport, the difference in transport time was added to the neurointerventionalist arrival to groin puncture time of 20 min to simulate a delay or acceleration in the arrival of the neurointerventionalist to the PSC. For DS transport, the difference in transport time was added to the door to groin puncture time at the CSC/CSC+ to simulate a delay or acceleration in the arrival of the patient to the CSC/CSC+. For mothership transport the difference in transport time was added to both the door to needle and door to groin puncture time at the CSC and CSC+ to simulate a delay or acceleration in the patient arriving at the CSC/CSC+.

### Map Generation

Study results were visualized using DESTINE mapping software (DESTINE Health Inc., Calgary, AB, Canada). To indicate which transport option predicts the best probability of good outcome the maps are color coded: red areas indicate DS predicts the best probability of good outcome, green areas indicate mothership predicts the best probability of good outcome, and purple indicates DD predicts the best probability of good outcome. Areas with no road infrastructure are marked gray. All PSCs are depicted as yellow pinpoints, all CSCs are depicted as light blue pinpoints, and all CSC+ as dark blue pinpoints.

### Ethics Statement

As no patient data were used for this study, institutional review board approval was not sought.

## Results

**Fig. 1:**
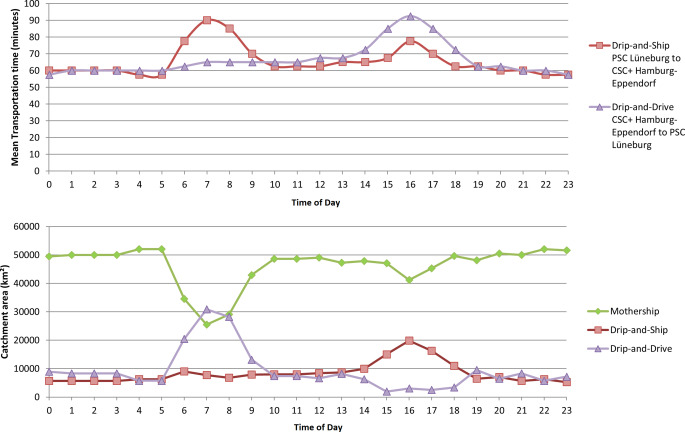
Mean transportation time (*upper panel*) and catchment areas (*lower panel*) of paradigms on weekdays. Shown is the diurnal mean transportation time and the catchment area in km^2^ of the 3 paradigms weekdays given a total area of 64,065 km^2^

**Fig. 2:**
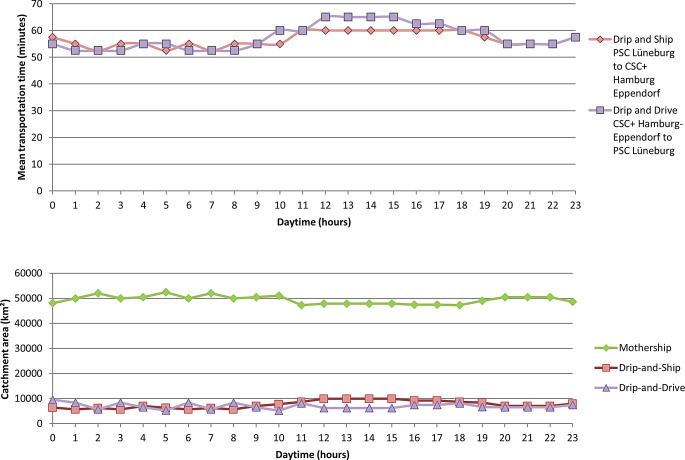
Mean transportation times (*upper panel*) and Catchment areas (*lower panel*) of paradigms on the weekend. Shown is the diurnal mean transportation time and the catchment area in km^2^ of the 3 paradigms on the weekend given a total area of 64,065 km^2^

Mean transportation times and the catchment areas of the three paradigms during weekdays are shown in Fig. 1 and on the weekend in Fig. 2.

**Fig. 3:**
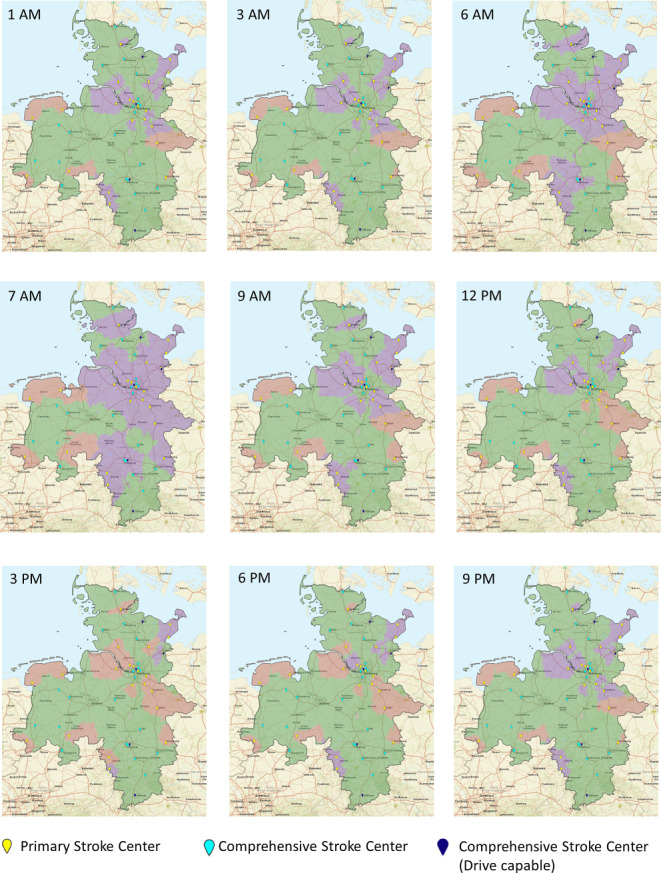
Diurnal modeled transportation option maps in northwest Germany at weekdays. Red areas indicate drip-and-ship paradigm predicts the best probability of good outcome, green areas indicate mothership paradigm predicts the best probability of good outcome, and purple indicates drip-and-drive paradigm predicts the best probability of good outcome

**Fig. 4:**
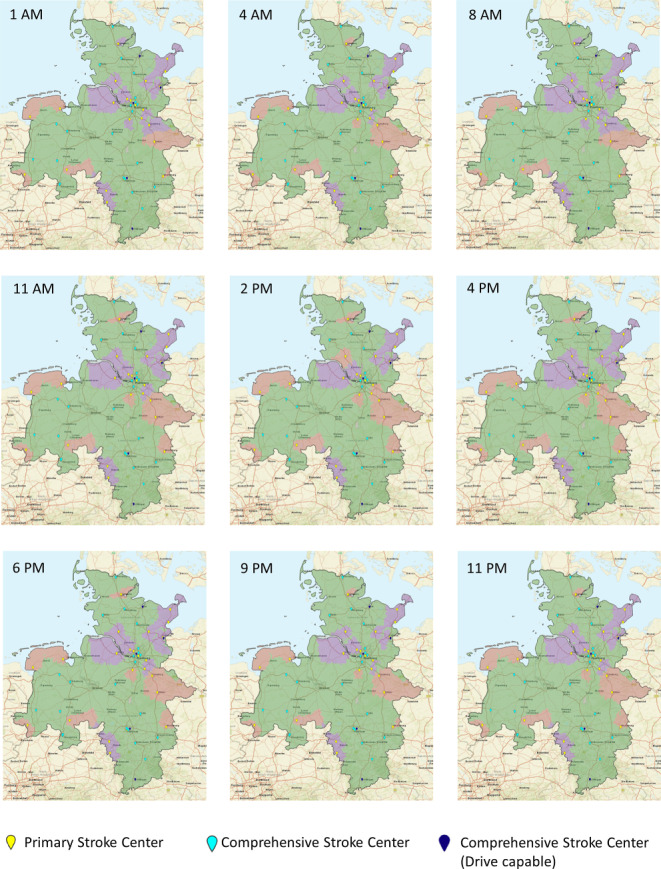
Diurnal modeled transportation option maps in northwest Germany on the weekend. Red areas indicate drip-and-ship paradigm predicts the best probability of good outcome, green areas indicate mothership paradigm predicts the best probability of good outcome, and purple indicates drip-and-drive paradigm predicts the best probability of good outcome

Diurnal modeled transportation option maps in northwest Germany during weekdays are shown in Fig. 3 and on the weekend in Fig. 4 and can be viewed as a diurnal movie in the online only data supplement.

On weekdays, the mean transportation time from the urban to the suburban area was 66 min. It was less or equal to 66 min from 7 p.m. to 11 a.m. Peak traffic period occurred from 3 a.m. to 5 p.m. with a maximum of 93 min at 4 p.m. The mean transportation time from the suburban to the urban area was 65 min. It was less or equal to 65 min from 6 p.m. to 5 a.m. Two peak traffic periods occurred from 6 a.m. to 9 a.m. with a maximum of 90 min at 7 a.m. and from 3 p.m. to 5 p.m. with a maximum of 78 min at 4 p.m. Mothership predicted the best outcomes in the majority of the region (≥40,000 km^2^, 63%) during the entirety of the weekday with exception of the morning rush hour at 7 a.m. where the mothership catchment area decreased to 25,467 km^2^, (40%). The DD catchment area peaked at 7 a.m. (30,879 km^2^, 48%) and was lowest from 3 p.m. to 6 p.m. having a catchment area of only 3–5%. The DS catchment area peaked with 31% at 4 p.m.

On weekends, the mean transportation time from the urban to the suburban area was 57 min. It was less or equal to 57 min from 8 p.m. to 9 a.m. It showed a maximum of 65 min from 12 p.m. to 3 p.m. The mean transportation time from the suburban to the urban area was 57 min. It was less or equal to 57 min from 7 p.m. to 10 a.m. It showed a maximum of 60 min from 11 a.m. to 6 p.m. Mothership predicted the best outcomes in the majority of the region (≥47,200 km^2^, 74%) during the entirety of the weekend but was lowest from 10 a.m. to 6 p.m. From 1 a.m. to 10 a.m. the DD and DS catchment areas were approximately 5205 km^2^ (8%) and 8376 km^2^ (13%) respectively with some hour over hour variation. From 9 a.m. to 11 p.m. the DS catchment area (11–15%) was larger than the DD catchment area (10–13%).

The number of EVT procedures performed by hour at the PSC Lüneburg are shown in online figure II in the online only data supplement. From 2016 to 2020, 128 emergency operations were performed, 106 (83%) on weekdays, 22 (17%) on weekends. The mean and median neurointerventionalist transportation time was 82 min both on weekdays and weekends. On weekdays, 69 (53%) emergency operations were performed between 8 a.m. and 5 p.m. Only 8 (6%) operations were performed from 12 p.m. to 7 a.m. There were no operations between 2 a.m. and 4 a.m. The maximum was performed at 10 a.m. with 15 (12%) operations. The transportation time was longest at 3 p.m. with a median of 102 min. On weekends, no operations were performed between 3 a.m. and 7 a.m., between 2 p.m. and 4 p.m., and at 8 p.m. The maximum was performed at 5 p.m. with 3 operations. Weekend transportation time was longest on a Saturday at 11 p.m. with a median of 95 min.

## Discussion

We have shown considerable variation of the catchment area for the different transportation paradigms for stroke patients across the course of the day both on weekdays and weekend. The findings are especially pronounced on weekdays. The mothership paradigm had the largest catchment area in northwest Germany 24h a day, 7 days a week with exception of the morning rush hour on weekdays. These results are consistent with previous modelling studies showing that PSCs in close proximity to an efficient CSC/CSC+ have to maintain short door to needle times to assure a comparable probability of good outcome [12, 13, 16, 17].

On weekdays, the predicted probability of good outcomes with the DD paradigm peaked at 7 a.m. and declined during the afternoon rush hour between 3 p.m. and 6 p.m. This can be explained by the fact that during morning rush hour the neurointerventionalist would be travelling in the opposite direction of the regular commuting traffic, and therefore encounters less road traffic congestion; however, during afternoon rush hour the neurointerventionalist is travelling in the same direction as the majority of commuters to the suburban areas during peak driving times, making the DD paradigm less advantageous; however, in the analysis of the data we found that the majority of EVT procedures performed at the PSC in Lüneberg occurred between 10 a.m. and 9 p.m., when travel time would be relatively low.

The DS paradigm was most advantageous during peak driving times on weekday afternoons. Similarly, the geographic context here is that the ambulance would be travelling from the suburban PSC to the urban CSC/CSC+ in the opposite direction of the regular commuting traffic during afternoon.

On Sundays, the mean transportation times from the urban CSC+ to the PSC and in the opposite direction were lower compared to weekdays and slightly increased in the afternoon, but did not show peak traffic periods.

The considerable impact of the diurnal variation in traffic rate and direction of travel found in this study provide compelling evidence that stroke systems of care should take time of day and real time traffic information into account when designing prehospital transport protocols. Including these estimated transportation times, could improve prehospital triage decisions and decrease treatment delay thereby improving clinical outcomes. This information could be implemented in mobile applications guiding the emergency medical services personnel to the right hospital.

Our observed mean neurointerventionalist transportation time of 82 min was longer than the predicted mean transportation time from the urban to the suburban area calculated with Google Maps (varying between 57–66 min). This is due to the fact that in our observational study the entire time from activation of the neurointerventionalist at the CSC+ at the CSC+ to arrival at the angiosuite of the PSC and not just their drive time. Recent studies have shown significantly shorter transfer times moving the neurointerventionalist from the CSC+ to the PSC in comparison with transporting the patient from the PSC to the CSC which resulted in shorter onset to EVT times [11, 18]. Moreover, the advantages of parallel processing in the DD paradigm have to be considered. During transfer of the neurointerventionalist from the CSC+ to the PSC, the patient can be transported to the angiography suite, prepared for the intervention, and intubated if necessary. The EVT times could be further reduced if local members of the radiology department started the intervention by placing the catheter sheath or even the guide catheter in the cervical arteries. As has been shown previously, DS and DD increase in favorability if treatment times are long at the CSC [12]. The DD paradigm enables sharing the workload and associated costs and hospital income between PSC and CSC+. The spread of procedure volume across multiple hospitals optimizes the use of beds for stroke patients throughout a region and allows adequate workload to maintain skill sets.

## Limitations

This work is not without limitations.

Firstly, the results of our model may not generalize to other cities. In areas where employment options tend to follow a more decentralized or polycentric model, there are more vehicles in the morning peak traffic period heading from downtown to the suburban area. Transportation times were calculated using Google Maps. Emergency medical services transportation times would be different, although it would not significantly influence the catchment areas, as all three paradigms would be affected in the same way; however, for the implementation of stroke treatment cooperation between CSC, CSC+ and PSC the local emergency medical services transportation times with diurnal variations in traffic rate and direction of travel should be assessed and considered.

Secondly, decay curves used in our model were derived from clinical trials containing highly selected patients in Europe and North America and might not be completely translatable to the general German population. Moreover, the percentage of patients treated with IVT or EVT influences the modeled results and might be different in other countries. The distance used for calculating the traffic time between the PSC and CSC+ was 68 km which is based on one PSC/CSC+ partnership in our system. While this is representative of the average distance between PSC and CSC+ in our system of 67 km, it likely overestimates travel time between PSC and CSC for DS (average distance of 44 km in our system). The generalization of the differences found for this 68 km transport were probably the least realistic for the mothership scenario as the majority of stroke patients are much closer to the CSC/CSC+.

Thirdly, several prehospital stroke severity scales have been developed to identify patients with LVO [19, 20]. In our model, we chose a score of ≥5 on the rapid arterial occlusion evaluation scale for the selection of the patient population. As has been shown before, catchment areas were similar using different screening tools [12], such as the Los Angeles motor scale [21] or the Cincinnati stroke triage assessment tool [22]; however, the results would be different with the development of a more accurate screening tool for LVO.

Finally, we did not consider air transport or mobile stroke units and effects of seasonality nor did we take into account out of the ordinary congestion, which can be the result of inclement weather, an accident, construction, or long holiday weekends.

## Conclusion

Using conditional probability modeling, our study showed a considerable impact of the diurnal variations in traffic rate and direction of travel on optimal stroke transportation. On weekdays, the mothership method had the largest catchment area except during morning rush hours, when the DD catchment area was highest. The DS catchment area was higher than the DD catchment area during the afternoon rush hours both during the week as well as on the weekend.

## Caption Electronic Supplementary Material

Online figure I: simulation of a delay or acceleration in transport time across the day for mothership, drip-and-ship, and drip-and-drive paradigms.

Movie 1. Diurnal transportation option maps in northwest Germany during weekdays. Red areas indicate drip-and-ship paradigm predicts the best probability of good outcome, green areas indicate mothership paradigm predicts the best probability of good outcome, and purple indicates drip-and-drive paradigm predicts the best probability of good outcome. The time of day is shown in the left upper corner.

Movie 2. Diurnal transportation option maps in Northwestern Germany during weekends*. *Red areas indicate drip-and-ship paradigm predicts the best probability of good outcome, green areas indicate mothership paradigm predicts the best probability of good outcome, and purple indicates drip-and-drive paradigm predicts the best probability of good outcome. The time of day is shown in the left upper corner.

Online figure II: number of diurnal emergency operations performed by CSC+ Hamburg-Eppendorf for PSC Lüneburg weekdays and on weekends.
