# Supplementary figures and images for: Modeling the Optimal Transportation for Acute Stroke Treatment: Impact of Diurnal Variations in Traffic Rate
Source: Clin Neuroradiol. 2020 Jul 16;31(3):729–36. doi: 10.1007/s00062-020-00933-y (PMC8463378; doi:10.1007/s00062-020-00933-y)

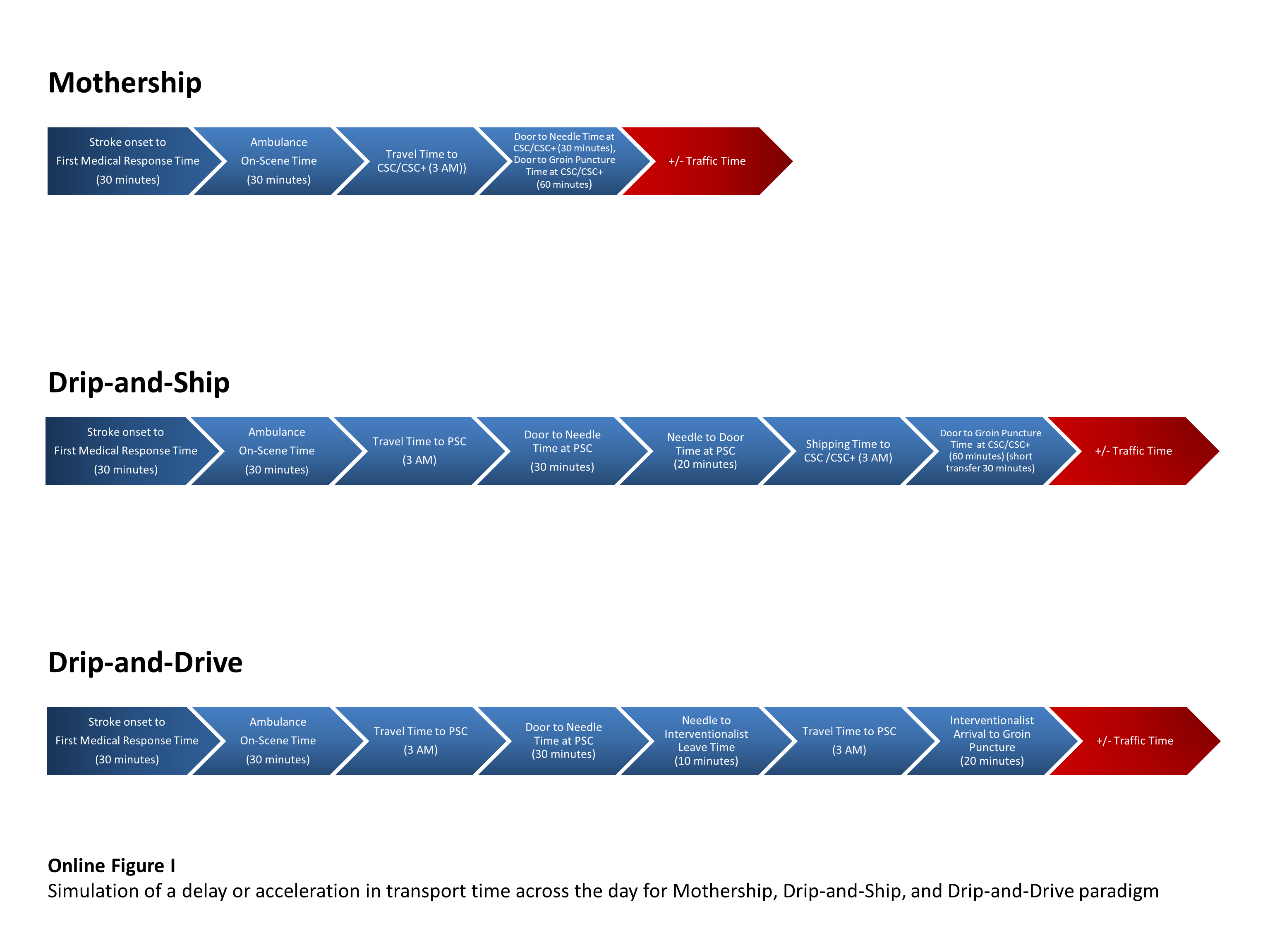

Supplement: Supplementary file 1 — Online figure I: simulation of a delay or acceleration in transport time across the day for mothership, drip-and-ship, and drip-and-drive paradigms. [file 62_2020_933_MOESM1_ESM.tif]

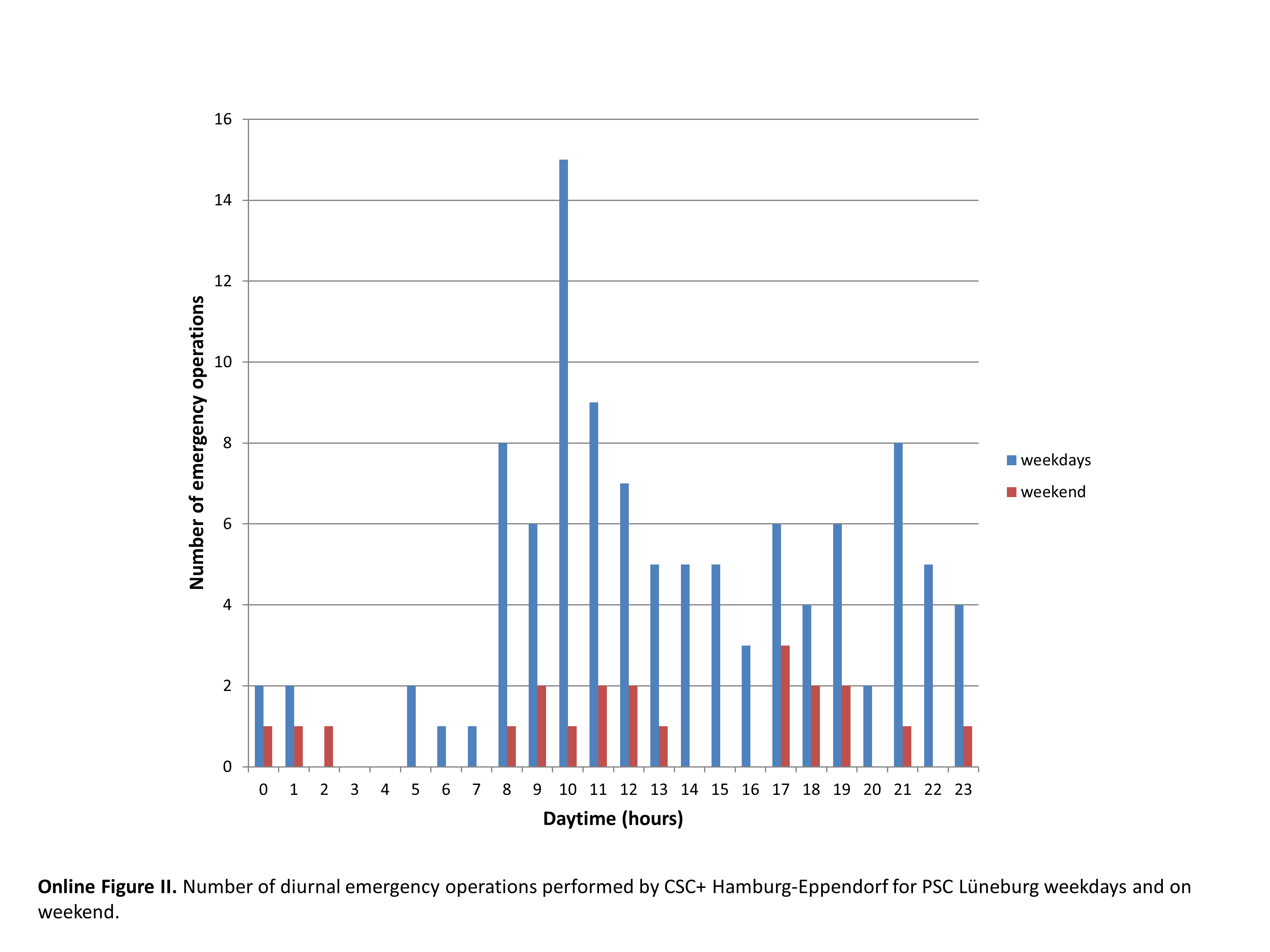

Supplement: Supplementary file 4 — Online figure II: number of diurnal emergency operations performed by CSC+ Hamburg-Eppendorf for PSC Lüneburg weekdays and on weekends. [file 62_2020_933_MOESM4_ESM.tif]
